# Optimizing Anti-PD1 Immunotherapy: An Overview of Pharmacokinetics, Biomarkers, and Therapeutic Drug Monitoring

**DOI:** 10.3390/cancers17193262

**Published:** 2025-10-08

**Authors:** Joaquim Faria Monteiro, Alexandrina Fernandes, Diogo Gavina Tato, Elias Moreira, Ricardo Ribeiro, Henrique Reguengo, Jorge Gonçalves, Paula Fresco

**Affiliations:** 1 Laboratório de Farmacologia, Departamento de Ciências do Medicamento, Faculdade de Farmácia, Universidade do Porto, 4050-313 Porto, Portugal; up202106588@edu.ff.up.pt (A.F.); up202107965@edu.ff.up.pt (D.G.T.); jgoncalves@ff.up.pt (J.G.); pfresco@ff.up.pt (P.F.); 2 UCIBIO, Unidade de Ciências Biomoleculares Aplicadas, Laboratório Associado i4HB, Instituto para a Saúde e Bioeconomia, Faculdade de Farmácia, Universidade do Porto, 4050-313 Porto, Portugal; 3 Serviços Farmacêuticos, Unidade Local de Saúde São João, 4200-319 Porto, Portugal; elias.moreira@ulssjoao.min-saude.pt; 4 Clinical Chemistry, Clinic of Genetics and Pathology, Centro Hospitalar de Santo António, 4099-001 Porto, Portugal or ricardo.ribeiro@i3s.up.pt (R.R.); henrique.reguengo.sqc@chporto.min-saude.pt (H.R.); 5 Tumour and Microenvironment Interactions Group, i3S Instituto de Investigação e Inovação em Saúde, Universidade do Porto, 4200-135 Porto, Portugal; 6 Unit for Multidisciplinary Research in Biomedicine (UMIB), Institute of Biomedical Sciences Abel Salazar (ICBAS), Universidade do Porto, 4050-313 Porto, Portugal

**Keywords:** anti-PD-1 drugs, cancer, pharmacokinetics, therapeutic drug monitoring, immune checkpoint inhibitors, biomarkers, clearance

## Abstract

**Simple Summary:**

The purpose of this review is to explore the therapeutic optimization of anti-PD-1 drugs by focusing on pharmacokinetic (PK) principles. The article discusses how interpatient variability and tumor burden affect drug exposure and highlights the PK–pharmacodynamic relationship in terms of exposure-efficacy and exposure-safety. It emphasizes the role of clearance variability, particularly baseline clearance and its dynamic reduction during treatment, as relevant biomarkers for stratifying patients and guiding individualized dosing strategies. The review also examines current dosing approaches, including fixed versus weight-based regimens, and emerging strategies such as therapeutic drug monitoring and PK-guided personalization. By integrating PK data, clearance biomarkers, and clinical outcomes, the review supports a shift toward more adaptive and personalized use of anti-PD1 therapies aimed at maximizing therapeutic benefit while minimizing adverse effects.

**Abstract:**

Anti-PD-1 therapies have transformed cancer treatment by restoring antitumor T cell activity. Despite their broad clinical use, variability in treatment response and immune-related adverse events underscore the need for therapeutic optimization. This article provides an integrative overview of the pharmacokinetics (PKs) of anti-PD-1 antibodies—such as nivolumab, pembrolizumab, and cemiplimab—and examines pharmacokinetic–pharmacodynamic (PK-PD) relationships, highlighting the impact of clearance variability on drug exposure, efficacy, and safety. Baseline clearance and its reduction during therapy, together with interindividual variability, emerge as important dynamic biomarkers with potential applicability across different cancer types for guiding individualized dosing strategies. The review also discusses established biomarkers for anti-PD-1 therapies, including tumor PD-L1 expression and immune cell signatures, and their relevance for patient stratification. The evidence supports a shift from traditional weight-based dosing toward adaptive dosing and therapeutic drug monitoring (TDM), especially in long-term responders and cost-containment contexts. Notably, the inclusion of clearance-based biomarkers—such as baseline clearance and its reduction—into therapeutic models represents a key step toward individualized, dynamic immunotherapy. In conclusion, optimizing anti-PD-1 therapy through PK-PD insights and biomarker integration holds promise for improving outcomes and reducing toxicity. Future research should focus on validating PK-based approaches and developing robust algorithms (machine learning models incorporating clearance, tumor burden, and other validated biomarkers) for tailored cancer treatment.

## 1. Introduction

While early-stage malignancies often respond favorably to conventional therapies, advanced cancers frequently exhibit resistance, requiring alternative treatment strategies. Cancer immunotherapy has transformed oncology by leveraging the immune system to target and eliminate tumor cells. The discovery of immune checkpoint regulation as a pivotal mechanism of tumor-immune evasion has revolutionized cancer treatment, leading to the development of novel immunotherapeutic approaches.

Immune checkpoints are regulatory proteins found on the surface of cells that help to maintain immune system balance by controlling the strength and duration of immune responses in lymphoid organs and peripheral tissues. This prevents autoimmunity and reduces tissue damage during inflammation. Well-characterized immune checkpoints include cytotoxic T-lymphocyte-associated antigen 4 (CTLA-4) and programmed cell death protein 1 (PD-1) and its ligands PD-L1 or PD-L2.

Concerning the PD-1/PD-L1 axis, PD-1 is a coreceptor that plays a role in the inhibition of T cell function [1,2]. When activated by its ligands, PD-1 switches off the cascades that cause T cell activation, inhibiting T cell function and contributing to T cell exhaustion [3,4,5], which is a state of cellular dysfunction where T cells become progressively less effective at performing their immune functions. PD-1 expression was primarily described on activated T cells [6], and its expression was described afterwards on B cells [7], dendritic cells (DCs) [8], macrophages [9], and natural killer (NK) cells [10].

PD-1 expression is downregulated under basal/resting conditions across multiple cell types, and its expression levels drastically increase within several hours after stimulation [11]. Upregulation of PD-1 and PD-L1 expression occurs during inflammation, acting to help to regulate and restrain the immune system in inflamed tissues [12]. In fact, PD-1 also plays a crucial role in maintaining the balance between effective immunity and self-tolerance, controlling self-reactive clones of immune cells and limiting autoimmunity [13,14].

Immune checkpoint inhibitors (ICIs) are monoclonal antibodies (mAbs) that block inhibitory regulatory pathways, thereby restoring T cell activation. In the context of inflammation, ICIs disable these systems’ capacity to restrain the immune system in inflamed tissues and to regulate peripheral tolerance. In the context of cancer, ICIs restore immune competence by reversing T cell exhaustion and restoring tumor antigen-specific cytotoxic T lymphocyte activity, promoting the elimination of malignant cells [15]. Therefore, these agents have become integral to first- and second-line treatment regimens for refractory tumors, although durable responses and survival benefits are limited to a subset of patients, reflecting ongoing challenges in identifying reliable predictors of response [16].

Key anti-PD-1 mAbs, including nivolumab, pembrolizumab, and cemiplimab have demonstrated clinical benefit across multiple malignancies, notably advanced melanoma, non-small cell lung cancer (NSCLC), and clear cell renal carcinoma [17]. Despite their transformative potential, anti-PD-1 therapies exhibit significant interpatient variability in both efficacy and toxicity. A considerable proportion of patients fail to derive meaningful clinical benefit, and others may develop acquired resistance. Furthermore, immune checkpoint blockade is associated with a variety of immune-related adverse events (irAEs), affecting over 25% of patients to which anti-PD-1 therapies contribute to around 10–15% of cases, with severe reactions in 6–10% and fatal outcomes reported in 0.3–1.3% of patients [18].

Given the high interindividual variability in response and the risk of serious toxicity, optimizing anti-PD-1 therapy requires careful pharmacokinetic (PK) and pharmacodynamic (PD) analysis. Ensuring adequate drug exposure is essential to achieving therapeutic efficacy while minimizing adverse effects. Additionally, understanding resistance mechanisms and identifying reliable biomarkers—such as tumor mutational burden (TMB), PD-L1 expression, the microbiome, hypoxia, interferon-γ signaling, and extracellular matrix composition—are critical to guide personalized treatment approaches [19].

This review aims to explore how the understanding of PK and PD and exploring therapeutic drug monitoring (TDM) can contribute to developing personalized dosing strategies and improving treatment outcomes. To conclude, we will consolidate the current knowledge and evidence base relating to the PKs, biomarkers, and monitoring of anti-PD-1 mAbs, with the ultimate goal of optimizing their clinical use. The review is organized into five sections: (i) the PK of anti-PD-1; (ii) PK-PD relations; (iii) available biomarkers for anti-PD-1 therapies; (iv) exploring PK and TDM to optimize dosing strategies; and (v) perspectives on translating PK insights into clinical practice and future research priorities.

## 2. Pharmacokinetics of Anti-PD-1

The commercially available anti-PD-1 drugs—pembrolizumab (Keytruda^®^, Merck Sharp & Dohme B.V., Haarlem, The Netherlands), nivolumab (Opdivo^®^, Bristol-Myers Squibb Pharma EEIG, Dublin, Ireland), and cemiplimab (Libtayo^®^, Regeneron Ireland Designated Activity Company, Dublin, Ireland)—are mAbs administered via intravenous infusion, resulting in complete bioavailability. However, alternative routes (e.g., subcutaneous administration) are under investigation to improve patient convenience. The PK of anti-PD-1 mAbs is conditioned by a complex interplay of factors related to the patient but also by tumor-specific factors. These factors include, but are not limited to, lymphatic and interstitial fluid dynamics, transendothelial movement of mAbs, genetic polymorphisms, immunogenicity, immune status, levels of endogenous IgG and albumin, co-morbidities, concomitant medications, and demographic factors such as age, sex, body size, and ethnicity [20], which give particularities to the PK of anti-PD-1 mAbs.

Anti-PD-1 therapies share similar PK profiles (Table 1), characterized by long half-lives, minimal hepatic metabolism, and time-dependent changes in PK, particularly in clearance (CL) mechanisms [21,22]. The temporal variability of CL of these agents warrants thorough consideration in this review. The underlying mechanisms and the factors already identified that contribute to this phenomenon will be comprehensively reviewed.

### 2.1. Disposition Mechanisms of Anti-PD-1 mAbs

The disposition of anti-PD-1 mAbs is governed by a combination of selective and non-selective mechanisms. Selective elimination occurs through target-mediated drug disposition (TMDD), driven by binding to PD-1 receptors on T cells. Non-selective pathways include interactions with gamma and neonatal Fc receptors, tissue proteolysis within the reticuloendothelial system, and the presence of circulating autoantibodies. In addition, the formation of anti-drug antibodies (ADAs) may alter both clearance and therapeutic efficacy. Ultimately, the PK and PD of anti-PD-1 therapies result from the complex interplay among these mechanisms, which together determine drug exposure, response, and variability across patients.

#### 2.1.1. Target-Mediated Drug Disposition

Anti-PD-1 mAbs are proteolytically degraded by systems that are independent of hepatic enzyme metabolism. Anti-PD-1 mAbs undergo proteolytic degradation intracellularly after a receptor-mediated internalization process occurring in cells express membrane “receptors” with affinity for these mAbs, contributing their CL. PD-1 is a protein found on the surface of cells that may undergo internalization, subsequent ubiquitination, and degradation by the proteasome in activated T cells [26]. PD-1 is the most obvious “receptor” for the anti-PD-1 mAbs and binding of anti-PD-1 mAbs to PD-1 can induce internalization of anti-PD-1/PD-1 complexes, and these complexes can be found in late endosomes [27] where they will be exposed to the proteolytic activity of the lysosomal enzymes. Therefore, the removal of surface PD-1 is both a critical step in the PD as it is the mechanism to remove PD-1 from the membrane, but also a critical step in the CL of the mAbs as it is a mechanism to promote the proteolytic degradation of the anti-PD-1 mAbs. The CL of anti-PD-1 mAbs may be closely linked to their PD effect, as CL can be seen as the result of the ‘heroic sacrifice’ after their PD action. Consequently, the CL of anti-PD-1 mAbs is enhanced when greater PD-1 receptor availability promotes increased binding, internalization, and degradation of the antibody–receptor complex. While internalizable PD-1 receptors are present, a linear CL of mAbs will predominate and elimination will be primarily driven by endocytosis and proteolytic degradation of the complex PD-1/anti-PD-1 mAb. Successful antitumor therapy should reduce the CL of the anti-PD-1 mAbs, since less tumor mass should mean fewer PD-1-positive cells, and, thus, fewer PD-1 receptors to which anti-PD-1 mAbs can bind, and fewer conditions allowing their metabolization.

Not all PD-1 receptors may be internalized. A fraction persists in the membrane during exposure to anti-PD-1 mAbs [27]. Therefore, the presence of PD-1 alone does not guarantee that mAbs will be effective or that the expected reduction in CL will be achieved over time. Several factors can contribute to this resistance to internalization, namely the density of PD-1 receptors or saturation of the endocytic machinery [27], level of PD-1 fucosylation [28] or its location in lipid rafts [29,30]. The impact of these factors on activity and CL may differ according to the mAbs used and may be altered by changes in tumor metabolism during treatment. Therefore, variations in the CL of anti-PD-1 mAbs over time must be interpreted with caution, as they may be influenced by the loss of the receptors’ ability to be internalized, resulting in tumor resistance to treatment.

#### 2.1.2. Non-Selective Mechanisms

##### Neonatal Fc Receptor

The available anti-PD-1 mAbs possess IgG4 subclass-specific Fc regions that specifically bind to Fc receptors (FcRs), presenting affinity to the neonatal Fc receptor (FcRn) which may also have impact on anti-PD-1 mAbs PK, although with consequences distinct from the binding to the PD-1 receptor.

FcRn is predominantly localized within the intracellular vesicular network, with less than 10% detected at the plasma membrane [31]. This observation should be interpreted with caution, as the relatively low proportion at the cell surface may reflect the receptor’s highly dynamic recycling behavior, analogous to that of the transferrin receptor [31,32]. FcRn protects its natural ligands (such as IgG antibodies and human serum albumin; ALB) from acidic proteolysis in endolysosomes [33,34]. The affinity of ligands to FcRn increases as pH decreases [35] and this pH dependency of FcRn to protect its ligands from proteolysis in the low pH found in the endolysosomes creates optimal conditions to maintain the FcRn/ligand complex protected from lysosomal degradation [36,37,38]. This, in turn, favors a recycling pathway of the anti-PD-1 mAbs back to the plasma membrane and its subsequent release from the cell. During this process, the ligands are preserved, which results in a prolonged serum half-life for ALB and IgG of about 20 days [39]. This capacity of FcRn to preserve ligands from proteolysis makes it a mechanism to promote transcytosis of IgG and ALB across epithelial barriers, both from basolateral to apical surfaces and vice versa in polarized cells [40].

FcRn is present throughout human tissues, with expression in endothelial and immune cells, being particularly expressed in a specialized manner at barrier sites, including the blood–brain barrier, and the placenta and mucosal surfaces [41]. This widespread distribution enables FcRn to serve as a universal recycling system through transcytosis of its natural ligands, IgG antibodies, and ALB, playing a key role in their homeostasis [36,42]. Biological drugs with Fc region are also FcRn substrates [43], including the anti-PD-1 mAbs [44,45]. Interaction of FcRn with anti-PD-1 mAbs increases the volume of distribution, reducing plasma concentration and prolonging their half-life.

The pH dependence of FcRn–ligand interactions have important consequences for the net outcome of FcRn-mediated transcytosis. At physiological pH (7.4), FcRn exhibits negligible binding to its ligands, whereas strong binding occurs at acidic pH values of 6.0–6.5 [32]. Consequently, under normal physiological conditions, the pH gradient between the compartments in which transcytosis may be occurring is negligible, without alterations in FcRn affinity caused by the pH. Consequently, the net transfer of ligands will be determined by their availability in the different compartments. In certain pathological conditions, such as cancer, the pH of the tumor microenvironment can be as low as 5.5 [46,47]. Such pH gradient creates an additional variable because it will change the affinity of FcRn and, by consequence, its capacity to release its ligands (including the anti-PD-1 mAbs) in the tumor microenvironment in comparison to normal tissues. Consequently, there will be a reduction in the conditions for transfer and retention of the drug in the acidic tumor microenvironment, which may result in a decrease in treatment efficacy.

##### Gamma Fc Receptor

Fcγ receptors (FcγRs) constitute a heterogeneous family of receptors that differ in their affinity for IgG subclasses, cellular distribution, and immunological functions. They are broadly categorized into activating receptors (FcγRI, FcγRIIa, FcγRIIIa) and the inhibitory receptor FcγRIIb [48]. FcγRI binds IgG with high affinity and is predominantly expressed on monocytes, macrophages, and DCs, mediating antibody-dependent cellular cytotoxicity (ADCC) and phagocytosis [48]. FcγRIIa and FcγRIIIa, with intermediate to low affinity, are expressed on macrophages, neutrophils, and NK cells, contributing to cytokine release and cytotoxicity [48]. In contrast, FcγRIIb is the only inhibitory FcγR, expressed on B cells, macrophages, and DCs, acting as a negative regulator of immune activation [49]. In addition to its inhibitory role, FcγRIIb participates in the clearance of antibodies by mediating endocytosis and lysosomal degradation in FcγRIIb-expressing cells, including liver sinusoidal cells and macrophages [49,50].

Although most approved anti-PD-1 antibodies are IgG4 isotypes carrying the S228P mutation, they are designed to minimize effector functions while retaining some binding to FcγRI and FcγRIIb [48]. Importantly, FcγR interactions have also been associated with hyperprogression during PD-1 blockade. Several clinical studies documented higher rates of hyperprogression in patients treated with anti-PD-1 mAbs compared to chemotherapy [51,52,53]; Lo Russo et al. specifically linked this phenomenon to FcγR^+^ macrophage activity in NSCLC [54] and it may also involve the proliferation of highly suppressive tumor-infiltrating PD-1^+^ effector Treg cells, which inhibit antitumor immunity and contribute to hyperprogressive disease [55,56].

Beyond these functional effects, FcγR binding may also contribute to the elimination of anti-PD-1 mAbs. Non-specific clearance of anti-PD-1 mAbs occurs through proteolysis in the liver and the reticuloendothelial system, particularly via FcγR-expressing cells such as Kupffer cells, monocytes, and macrophages [57]. Following receptor-mediated endocytosis, antibodies are degraded in lysosomes. Although this pathway is generally nonsaturable and therefore unlikely to markedly alter the pharmacokinetics of anti-PD-1 mAbs when non-specific CL predominates, FcγR-mediated elimination can interact with pharmacodynamic mechanisms and may indirectly accelerate CL, as shown for rituximab [58].

Together, these findings suggest that FcγR binding undermines both the pharmacodynamics and, to some extent, the pharmacokinetics of anti-PD-1 antibodies.

##### Tissue Proteolysis

Anti-PD-1-mAbs are also substrates of matrix metalloproteinases (MMPs) that may potentially affect their PK and PD. MMP-13 and MMP-2 are the metalloproteinases reported to be involved in anti-PD-1 mAbs proteolysis [59]. Cleavage of anti-PD-1 mAbs formed the fragments F(ab’)_2_ and Fc. Concerning the F(ab’)_2_ fragment, although it retains the antigen binding site, it has been reported as less active in inducing T cell proliferation than the respective anti-PD-1 mAbs [60], and inactive in removing PD-1 from the immune synapse and in preventing tumor growth in vivo [61]. Fc fragments at CH2 formed after protease action may retain the capacity to bind to FcRs and to compete with its ligands and, therefore, it is likely that Fc fragments may bind to FcRn and competitively inhibit the binding of other FcRn ligands (including anti-PD-1 mAbs), favoring the lysosomal degradation pathway and decreasing the half-life of anti-PD-1 mAbs [62,63]. Therefore, due to the activity of MMP, it is plausible to observe alterations in the PD-PK of anti-PD-1 mAbs, including reduced therapeutic efficacy due to decreased affinity for PD-1 and a shorter half-life resulting from diminished binding to FcRn.

MMP-13 and MMP-2 are not ubiquitously expressed: MMP-13 is a collagenase that plays a fundamental role in extracellular matrix (ECM) remodeling, particularly in bone biology. MMP-2 is also involved in ECM remodeling and is implicated in tissue repair, wound healing, angiogenesis, embryogenesis, bone remodeling, and tooth development [64]. Both have been found to be overexpressed in various types of cancer: MMP-13 in breast cancer, head and neck carcinomas, chondrosarcomas, and skin carcinomas [65]; MMP-2 in breast cancer [66], ovarian [67], oral [68], prostate [69], bladder [70], colorectal [71] cancers, retinoblastoma [72], and laryngeal squamous cell carcinoma [73]. Expression of both MMP-13 and MMP-2 has been associated with aggressive or invasive tumors and with poor clinical outcomes [74,75], as well as with various cell types present in the tumor microenvironment, including tumor-associated macrophages [76,77,78] and cancer-associated fibroblasts [64,79,80]. As these cell populations increase in the tumor microenvironment, the conditions for the proteolysis of anti-PD-1 mAbs within the tumor microenvironment will also increase, thus favoring the development of resistance to these ICIs.

##### Autoantibodies

Pathogenic autoantibodies are high-affinity, somatically mutated IgG autoantibodies that are stimulated by inflammation in the target organ [81,82]. These IgG autoantibodies are also FcRn ligands, which means interaction between FcRn and the autoantibodies are the key to removing them from inflamed tissue [83]. Sharing the same transcytosis pathway, FcRn may represent a potential site of interaction between anti-PD-1 mAbs and IgG autoantibodies, which can have clinical implications. In the tumor microenvironment, the pH in areas of inflammation is also in the acidic range [84], so the affinity of FcRn for the ligands remains high, which means there are less conditions to release the ligands carried to the areas of inflammation and less conditions for the ligands formed in the areas of inflammation to bind to FcRn. When anti-PD-1 mAbs are used, these exogenous ligands contribute to saturating FcRn transport in a dose-dependent manner and competing with IgG autoantibodies for transport out of the inflamed zone. This increases the risk of aggravating inflammation and irAEs, as previously reported [85,86,87,88,89].

#### 2.1.3. Anti-Drug Antibodies

The PD and PK of therapeutic proteins such as mAbs may be influenced by antibodies that are formed in response to the drug (anti-drug antibodies-ADAs) which have the potential to alter the efficacy of the treatment. Some ADAs are neutralizing antibodies, which means they block or inactivate the drug’s therapeutic effect; others may simply increase the drug’s clearance from the body (clearing antibodies), reducing drug exposure [90]. In the case where anti-PD-1 mAbs are approved, the risk to produce ADAs seems to be higher with nivolumab (occurrence in about 11% of patients) [91], although without clinical relevance [92]. Nevertheless, the production of ADAs is a factor that should be monitored since its incidence increases when anti-PD-1 mAbs are used in combination therapies [91].

### 2.2. Pharmacokinetic Models

**Figure 1:**
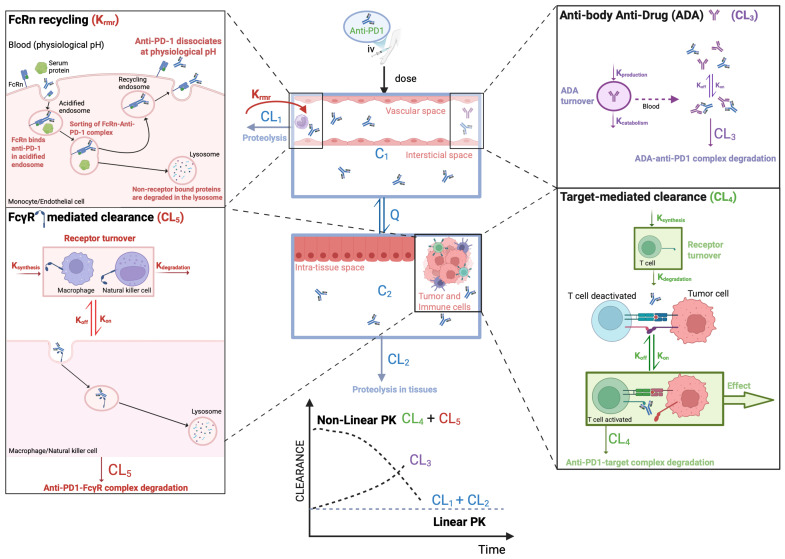
Anti-PD1 pharmacokinetic model to estimate time-dependent changes in clearance (CL). Legends: ADA—antibody anti-drug; K_synthesis_—synthesis constant; K_degradation_—degradation constant, K_off_—dissociation constant; K_on_—association constant, iv—intravenous. Figure elaborated using BioRender.com.

A putative PK model integrating all these mechanisms and their interrelationships is illustrated in Figure 1. The CL of anti-PD-1 mAbs can be described through a combination of linear and non-linear disposition mechanisms. Linear CL predominates when the PD-1 receptor is saturated, and elimination is primarily driven by proteolytic catabolism in plasma and peripheral tissues. In contrast, TMDD—a mix of linear and non-linear CL—occurs when the receptor is not saturated. In this scenario, elimination is governed by both proteolytic catabolism and receptor-mediated endocytosis from the plasma or interstitial into target cells [93,94]. In addition, the FcRs play a central role in regulating the half-life of anti-PD-1 antibodies through recycling mechanisms that prevent lysosomal degradation and/or elimination mechanisms. Interindividual variability in FcRs function—affected by factors such as systemic inflammation, hypoalbuminemia, or competing endogenous IgG—can influence drug CL and contribute to differences in drug exposure. Furthermore, the development of anti-drug antibodies (ADAs) may enhance antibody CL by forming immune complexes that are rapidly eliminated.

This variability in CL has clinical implications, particularly over time, as it contributes to the progressive increase in drug exposure observed with repeated dosing. This effect is more pronounced in responders compared to non-responders [24,94,95,96], and it will be discussed later.

### 2.3. Factors Influencing Pharmacokinetics of Anti-PD-1

#### 2.3.1. Albumin and Cachexia

Albumin (ALB) has emerged as a significant covariate influencing the PK of anti-PD-1 mAbs, particularly pembrolizumab and nivolumab. Across multiple studies, lower baseline ALB levels have been consistently associated with increased CL of both agents, underscoring the clinical importance of the patient’s nutritional status for therapeutic antibody disposition. In patients treated with pembrolizumab, those with higher CL exhibited significantly worse overall survival (OS), especially in patients with NSCLC and malignant pleural mesothelioma [97]. This relationship can be partly explained by the finding that patients with lower ALB levels before treatment demonstrate faster elimination of pembrolizumab, while higher ALB levels are associated with reduced CL [97]. Similarly, in patients receiving nivolumab, a strong and clinically meaningful association between ALB and drug CL has been observed. ALB levels exerted a greater than 20% effect on CL, with patients presenting hypoalbuminemia experiencing significantly higher nivolumab elimination [98].

A possible mechanism could be the interaction with FcRn; although ALB and IgG molecules are substrates of FcRn, they bind to distinct sites on the receptor [99,100]. Nevertheless, they may still compete for the overall availability of FcRn [101,102]. Consequently, hypoalbuminemia could increase FcRn availability for IgG molecules, indirectly affecting their homeostasis. An identical interaction may also occur with anti-PD-1 mAbs with potential clinical impact.

Performance status (PS), another indicator of disease severity, has been shown to correlate with ALB, where patients with poorer PS present lower ALB levels, thereby potentially influencing CL indirectly [98]. Several mechanisms are likely involved, being hard to discriminate but, overall, these findings emphasize the need to consider ALB levels and cachexia-related markers when optimizing ICIs dosing.

Cachexia negatively influences patients’ outcome during anti-PD-1 therapy [103] which is associated with reduced systemic concentrations of therapeutic antibodies [104], and elevated CL of anti-PD-1 mAbs parallel with a whole-body protein turnover observed in this population [105], which may contribute to reduced efficacy and poor clinical outcomes. Hypoalbuminemia has been used as a biomarker for cachexia but low ALB cannot explain the elevated CL of anti-PD-1 mAbs since it would be expected that low ALB would provide better conditions for anti-PD-1 mAbs to bind to FcRn, and thus, be spared from target-mediated CL and other forms of non-selective CL. Cachexia has shown to reduce FcRn expression [106]. Thus, from a reduction in the participation of FcRn in anti-PD-1 mAbs homeostasis, an acceleration of the mAbs degradation is expected to occur, with a marked increase in CL.

Low ALB levels may not only signal poorer prognosis but also suggest PK adjustments to maintain therapeutic drug exposure. Moreover, reductions in anti-PD-1 CL during therapy may potentially serve as a surrogate marker for clinical improvement or attenuation of cachexia.

#### 2.3.2. Disease Burden

PK studies of anti-PD-1 mAbs have consistently demonstrated time-varying CL, which is closely associated with disease burden [24,107]. These studies reveal a progressive decline in CL over time, particularly in patients who exhibit a reduction in tumor mass. This indicates that tumor burden is a dynamic and influential covariate in the disposition of anti-PD-1 therapies [24], altering factors that selectively or non-selectively influence the PK of anti-PD-1 mAbs. For example, patients with higher tumor burden and more advanced disease, who are more likely to be cachectic, may exhibit a higher monoclonal antibody CL. Conversely, regression of tumor burden through effective treatment may alleviate cachexia, reduce protein catabolism, and lead to reduced antibody CL [93].

These findings highlight the importance of considering tumor dynamics when interpreting and trying to personalize anti-PD-1 treatment. This will enable us to better interpret the real-world responses to these drugs, develop more accurate biomarkers with a greater predictive capacity of efficacy, failure, and toxicity, and develop treatment algorithms to improve the effectiveness of anti-PD-1 mAbs.

## 3. Pharmacokinetic–Pharmacodynamic Relations

### 3.1. Exposure–Efficacy Relations

Dynamic changes in the PK of anti-PD-1 mAbs, particularly time-dependent reductions in CL, have been increasingly recognized as important correlates of clinical efficacy. Patients with clinical response tend to experience a marked decrease in CL over time, indicating increased systemic drug exposure during treatment. This phenomenon is particularly evident in patients achieving complete response, who often show the greatest reductions in CL—around 45% in the case of nivolumab—compared to lower reductions in partial responders (35%), those with stable disease (25%), and patients with progressive disease (20%) [21,24]. For pembrolizumab, longitudinal PK data have shown that patients stratified by quartiles of decreasing CL exhibited significantly improved response rates and overall survival, even if they began treatment in worse clinical conditions such as cachexia [24]. This supports the notion that exposure to anti-PD-1 increases, driven by reduced CL, can be both predictive and prognostic indicators of benefit. Importantly, these PK improvements appear to coincide with improved PS, which itself is a marker of overall patient condition and treatment response. Together, these observations reinforce that changes in drug exposure over time—rather than static early PK values—are more informative of the efficacy of anti-PD-1 agents.

Despite this, earlier pharmacometric studies for pembrolizumab and nivolumab suggested a flat exposure–response (E–R) relationship, especially when analyzing fixed-dose regimens. PD-1 receptor occupancy modeling demonstrated full saturation at clinically relevant doses, with no observed increase in efficacy beyond a certain exposure threshold. This led to the assumption that standard dosing regimens already sit at the plateau of the E–R curve, reducing the utility of TDM or individualized dose titration based on early exposure metrics [24,98,108]. However, more recent data challenge the flat E–R paradigm. Studies such as the one by Desnoyer et al. (2020) presented that CL—both at baseline and in its reduction over time—was the strongest predictor of response and survival in patients treated with anti-PD-1 drugs [21]. This suggests that exposure does matter, especially when considering the dynamic evolution of drug disposition during therapy. Moreover, while initial exposure levels (such as first-dose area under curve (AUC) or serum concentration) may not predict long-term outcomes, the evolving PK profile, particularly decreasing CL, correlates with clinical improvement and may justify exploring alternative dosing strategies tailored to individual patient response. For instance, patients demonstrating substantial reductions in CL might sustain efficacy with extended dosing intervals, which is a strategy that could improve patient convenience and reduce healthcare costs [24,109]. Adding further complexity, the apparent lack of E–R correlation at early timepoints may stem from the immunomodulatory nature of anti-PD-1 drugs. These agents restore T cell function rather than directly attacking tumor cells, and their activity can persist even after serum levels drop below quantifiable levels, due to sustained PD-1 receptor occupancy and immune memory effects [21,110,111,112,113].

These findings advocate for integrating longitudinal PK into clinical assessment, as CL dynamics may provide valuable insights into patient response and offer a pathway toward personalized dosing strategies in immunotherapy.

### 3.2. Exposure–Safety Relations

While reduced CL of anti-PD-1 mAbs is widely regarded as a favorable PK indicator associated with improved clinical efficacy, this elevated exposure may increase the risk of toxicity, particularly irAEs.

Patients treated with pembrolizumab—for example, those with marked reductions in CL, who are often complete responders—exhibit sustained high plasma drug concentrations, which could predispose them to heightened toxicity [114]. As tumor burden declines and fewer PD-1 receptors are available, TMDD of these drugs decreases. This leads to an accumulation of unbound antibody in circulation, further amplifying systemic exposure, particularly in patients with robust antitumor responses. Such elevated exposures may not always be entirely innocuous. Case reports have linked pembrolizumab to serious and sometimes fatal toxicities, including pericardial effusion [115], myasthenia gravis [116], and nephrotic syndrome [96]. While nephrotic syndrome and hypoalbuminemia generally increase mAbs CL, the resolution of this condition during effective pembrolizumab treatment indicates a complex interplay between disease burden, protein metabolism, and drug disposition. Still, the potential for pembrolizumab to contribute to or worsen renal injury remains a clinically relevant concern, especially given the known association of ICIs with interstitial nephritis. To mitigate toxicity, particularly in patients with marked CL reduction, PK-informed dose adaptation strategies are emerging. For instance, in patients showing >40–50% decrease in CL, extending the dosing interval may preserve efficacy, while lowering cumulative exposure may potentially reduce the risk of irAEs [117,118]. These strategies can enable safer and more personalized treatment regimens. Nonetheless, early dose–toxicity analyses often reported no clear exposure–toxicity relationship for anti-PD-1 agents. For nivolumab, findings have been similarly mixed. Although early exposure measures such as the average concentration after the first dose were not significantly associated with adverse events or treatment discontinuation, baseline CL was strongly predictive of both efficacy and survival [24]. Importantly, while higher exposure due to lower CL has not been consistently linked with increased toxicity risk of anti-PD-1 drugs, rare but severe late-onset irAEs such as thrombocytopenia and autoimmune endocrinopathies have been documented [119]. These may occur independently of serum drug levels, underscoring the complex, delayed, and immune-mediated nature of toxicities associated with PD-1 blockade. In early-phase trials, including those in melanoma, PD-1 occupancy in peripheral lymphocytes was saturated at relatively low doses (≥0.3 mg/kg), with little evidence of a dose-dependent increase in irAE risk [120]. Some exploratory studies even noted negligible differences in irAE incidence across various dosing regimens, including fixed-dose schedules, suggesting that PD-1 blockade achieves a therapeutic ceiling beyond which toxicity does not proportionally escalate [121,122]. However, more profound analyses complicate this narrative. In some patients, particularly those treated with 480 mg Q4W, a modest increase in grade 2 irAEs was noted compared to 3 mg/kg Q2W, when peak concentration was considered as the exposure variable [121]. This suggests that while trough levels may remain within therapeutic range, transient high exposures could still contribute to irAE onset. Furthermore, patients with high baseline levels of pro-inflammatory cytokines such as IFN-γ, IL-2, and TNF-α were more likely to develop irAEs, implying a host-related immunologic predisposition to toxicity [119]. Likewise, increased effector T cell counts prior to the onset of irAEs may indicate a pre-existing state of immune activation that becomes dysregulated upon checkpoint blockade.

In conclusion, while reduced CL and elevated exposure to anti-PD-1 therapies are desirable from an efficacy standpoint, they also carry an increased risk of toxicity in some patients. The absence of a consistent dose–toxicity relationship across the general population may reflect the immunologically driven nature of irAEs, which depend not only on drug exposure but also on host immune status, T cell repertoire, and cytokine milieu. These nuances highlight the need for individualized monitoring strategies that consider both PK parameters and immune profiling to optimize safety while preserving efficacy in anti-PD-1 treatment.

### 3.3. Clearance–Efficacy and/or Safety Relationships

**Figure 2:**
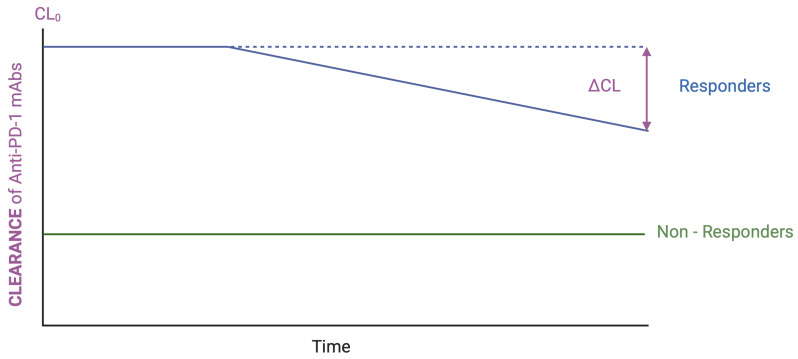
Anti-PD1 time-dependent changes in clearance and relation to treatment response. Legends: CL_0_—basal clearance; ΔCL—change in CL over time. Figure elaborated using BioRender.com.

In the PK evaluation of anti-PD-1 mAbs, two CL-based indicators have gained increasing recognition for their prognostic and pharmacodynamic relevance: baseline CL (CL_0_) and change in CL over time (ΔCL) [123]—Figure 2. CL_0_ refers to the initial estimate of drug CL typically calculated from plasma concentrations measured after the first or two doses. It reflects the patient’s initial capacity to eliminate the drug and can be influenced by both physiological and pathological factors, including TMDD, systemic inflammation, and cachexia. ΔCL describes the temporal variation in CL during treatment, most often expressed as the percent reduction from baseline. A decreasing ΔCL over time generally indicates an improving physiological or disease state, potentially corresponding to reduced antigen load, decreased inflammation, or resolution of cachexia. Together, CL_0_ and ΔCL offer valuable insights into the biological status of the patient and can act as dynamic biomarkers for treatment efficacy and outcome.

CL_0_ has emerged as a particularly strong prognostic marker. Studies of pembrolizumab, nivolumab, and other ICIs confirmed that CL_0_ is a dose-independent predictor of survival and response [44,124]. Turner et al. demonstrated that patients in the lowest quartile of pembrolizumab CL_0_ had significantly improved survival compared to those in the highest quartile, regardless of receiving 2 mg/kg or 10 mg/kg, illustrating that higher exposure could not compensate for high baseline clearance [124]. These findings are reinforced by flat E–R relationships observed within clinically relevant dosing ranges. For instance, objective response rate did not increase with pembrolizumab doses from 2 to 10 mg/kg, suggesting the lowest evaluated dose lies near the efficacy plateau [125]. Similarly, no meaningful correlation between drug concentration (Cmin) and efficacy was found for nivolumab over a broad dose range [92,126]. These observations highlight a frequent misinterpretation in pharmacometrics: when only a single dose level is studied, apparent E–R relationships may reflect CL–response (CL–R) correlations because exposure (AUC) and CL are inversely related at a constant dose. Thus, only by evaluating multiple dose levels with balanced patient characteristics can true E–R and CL–R relationships be distinguished [127,128]. Turner et al. emphasized this by showing steep E–R trends within dose strata (2 mg/kg or 10 mg/kg) but there was no relationship between the two groups, despite a 5-fold exposure difference [124]. This underscores the clinical relevance of distinguishing between E–R and CL–R: a true E–R relationship implies that dose escalation might improve outcomes, whereas a CL–R relationship suggests that increasing exposure will not alter response, and efforts should instead focus on understanding the biological basis of elevated clearance.

The mechanisms underlying high CL_0_ are multifactorial. High target expression and antigen load may drive TMDD, while systemic inflammation and cachexia—commonly present in advanced cancers—contribute to elevated catabolic clearance [44,124], resulting in responders exhibiting higher CL_0_ values compared to non-responders (see Figure 2). Conversely, a significant decline in ΔCL typically reflects treatment response and improvement in the disease status. Liu et al. quantified this association, showing that patients with CR to nivolumab exhibited a 42.4% reduction in CL, compared to 35.3%, 23.8%, and 20.1% for partial response, stable disease, and progressive disease, respectively [112]. Similar ΔCL–response associations have been observed across multiple ICIs, including pembrolizumab [129] and cemiplimab [130], suggesting a class-wide phenomenon. These observations provide further evidence that changes in CL dynamics are not merely PK artifacts but reflect underlying disease biology.

In contrast to its prognostic value for efficacy, the relationship between anti-PD-1 CL and irAEs remains poorly defined. Some single-center studies found no significant difference in CL between patients experiencing low-grade versus high-grade irAEs [97,131]. These inconsistencies highlight the need for prospective studies exploring the mechanistic underpinnings of CL pathways, including FcRs-mediated processes and their relationship to immune toxicity profiles.

In conclusion, CL_0_ and ΔCL are robust, exposure-independent PK biomarkers that predict clinical outcomes in patients treated with anti-PD1. Recognition of these CL–R relationships not only inform patient prognosis but also prevents misinterpretation of E–R data and the unnecessary pursuit of higher dosing strategies. Moreover, a deeper understanding of the biological factors driving elevated CL may uncover new therapeutic targets or stratification tools. While the link between CL and safety outcomes remains less clear, it warrants further mechanistic exploration to optimize treatment for vulnerable patient subpopulations.

## 4. Biomarkers for Anti-PD-1 Therapies

Despite notable clinical successes, immunotherapy with anti-PD-1 agents elicits durable responses in only a subset of cancer patients. Many do not respond to treatment or relapse after an initial treatment response. Identifying and validating biomarkers that can stratify patients likely to benefit from anti-PD-1 therapy remains a critical area of research, as the development, regulatory approval, and clinical adoption of cancer immunotherapies heavily rely on such biomarkers [132,133,134].

A recent meta-analysis using data from 100 peer-reviewed studies and almost 19,000 patients revealed that PD-L1 immunohistochemistry, TMB (neoantigens), and multimodal biomarkers discriminated responders and non-responders better than random assignment [133]. Other biomarkers are related to the immune system (peripheral blood cell populations, cytokine profiles, tumor-infiltrating lymphocytes, microbiome signatures) (see Table 2). Although some biomarkers consistently performed better, heterogeneity in performance across cancer types was observed, meaning that additional research is needed to identify accurate and precise biomarkers for widespread clinical use. Given the complex and dynamic nature of tumor-immune system interactions, the use of a single biomarker is unlikely to consistently yield accurate predictions of therapeutic response. Instead, a multidimensional approach integrating tumor-intrinsic factors and immune-related features—including protein expression, genomic alterations, and transcriptomic profiles—is emerging as necessary to improve predictive accuracy [135]. Prior to clinical implementation, however, candidate biomarkers and associated technologies must undergo rigorous validation to establish their clinical relevance and utility [136]. The Society for Immunotherapy of Cancer’s, recognizing the complexities of this research, has provided expert guidance on biomarker identification, validation, and application in cancer immunotherapy [134,137,138,139,140,141].

Clinically validated biomarkers predictive of response to anti–PD-1 therapy include PD-L1 expression, microsatellite instability, and TMB [158], although many others are being investigated (Table 2).

Anti-PD-1 therapies, like other ICIs, disrupt immune self-tolerance, leading to irAEs that often resemble autoimmune diseases, including rheumatic, dermatologic, gastrointestinal, hepatic, and endocrine disorders [159,160]. Owing to the growing use of anti-PD-1 in oncology, clinicians will increasingly be confronted with common but also with rare irAEs. As such, awareness needs to be raised among professionals regarding the clinical presentation, diagnosis, and management of these toxicities [161]. Ongoing research continues to also investigate biomarkers predictive of anti-PD-1 toxicity to improve patient management (Table 2). The use of machine learning models incorporating validated biomarkers across different conditions may represent a promising strategy to further enhance prediction and guide personalized treatment approaches.

## 5. Rethinking Dosing Strategies for Anti-PD-1: The Role of TDM

ICIs and, in particular, anti-PD-1 transformed the oncology landscape, offering new therapeutic avenues by enhancing the ability of the host immune system to recognize and attack tumor cells. Unlike conventional anticancer agents that act directly on tumor targets, these drugs exert their effects indirectly through immune modulation, resulting in atypical patterns of tumor response and adverse effects [162]. Despite these advances, durable clinical benefit is achieved in only a subset of patients, highlighting the need for more personalized approaches to maximize efficacy and reduce immune-related toxicity.

Pharmacometrics offers a systematic approach for precision dosing through the integration of PK and PD data, facilitating the optimization of therapeutic regimens. In this context, TDM has been proposed as a tool to guide individualized treatment by ensuring adequate drug exposure at the site of action. However, the unique pharmacological properties of mAbs, such as non-linear PK, TMDD, and immunogenicity, represent challenges to conventional TDM approaches [163]. While TDM is routinely used for small-molecule drugs with narrow therapeutic indices, its application to mAbs in oncology remains limited and controversial. Unlike small molecules, mAbs often lack a well-defined maximum tolerated dose, and their enhanced tolerability can obscure dose–response relationships. Additional complexities arise from delayed PD responses, development of anti-drug antibodies, and evolving immune profiles in cancer patients [164,165].

Initial PK/PD studies with nivolumab suggested a flat exposure–response relationship, with low doses achieving over 70% PD-1 receptor occupancy in circulating T lymphocytes, which is well below approved therapeutic doses [110]. However, this assessment did not account for drug activity in the tumor microenvironment, where therapeutic engagement is most relevant [166]. More recent evidence from patients with NSCLC has demonstrated a correlation between trough concentrations of nivolumab and clinical response, suggesting that PK parameters may indeed influence treatment outcomes [167].

Currently, anti-PD-1 are administered using fixed dosing regimens but given their substantial interindividual PK variability and long half-lives, such strategies may not be optimal for all patients [168]. The observed variability may result in subtherapeutic exposure or heightened toxicity in some individuals, especially in the absence of clear therapeutic windows. This underscores the need for adaptive dosing strategies, including Bayesian- and population-based models, to tailor therapy more effectively [169].

Several population pharmacokinetic (PopPK) models have been developed to characterize the PK of anti-PD-1 mAbs used in the treatment of various cancers. In a recent systematic review, 14 PopPK studies involving anti-PD-1 antibodies, including nivolumab, pembrolizumab, cemiplimab, camrelizumab, and dostarlimab, across a broad range of solid tumors were analyzed. Most models adopted a two-compartment structure with time-varying CL, reflecting PK changes over the course of treatment, which often associated with therapeutic response. Authors concluded that current PopPK models show good predictive performance and provide a robust foundation for the individualization of anti-PD-1 therapy. However, they emphasize the need for further external validation and exploration of additional clinical and biological covariates that may support more precise treatment tailoring [168].

Despite these challenges, TDM has demonstrated clinical utility in other immunologic indications, such as anti-TNF-α therapies [170] and tyrosine kinase inhibitors [171], suggesting potential transferability to oncology. Technological advances in drug quantification—such as ELISA, homogeneous mobility shift assay, electrochemiluminescence immunoassay, and LC-MS/MS—are facilitating more accurate and standardized measurement of mAb concentrations. Still, it is critical to distinguish between free, total, and complexed drug forms, considering the cross-reactivity and specificity of assays used [164].

We propose a TDM methodology integrated with clinical evaluation and biomarker analysis, focusing on the longitudinal CL evaluation as a dynamic indicator of both therapeutic effectiveness and potential toxicity. Monitoring should prioritize steady-state trough concentrations (Cmin, ss) which can be quantified using immunoassays such as the Shikari^®^ ELISA kit (BioVision, AbCam, Cambridge, UK). Some authors, namely Sureda et al., proposed a Cmin, ss target of 57 μg/mL for nivolumab, for example [172]. Rather than relying solely on target therapeutic exposure thresholds, TDM should be interpreted in the context of the patient’s evolving clinical response. The incorporation of TDM for anti-PD-1 into routine clinical practice may—as CL decreases, often in association with a favorable clinical response—enable dosing adjustments overtime by reducing the dose or extending the dosing interval, while maintaining adequate drug levels. This adaptive strategy offers the potential for sustained clinical benefit (such as maintaining response and reducing the risk of irAEs) along with economic advantages, supporting a more efficient and personalized use of anti-PD-1 therapies. Ultimately, this strategy aligns with the paradigm of achieving an optimal biologic dose or optimal individualized dose, especially in scenarios where the maximum tolerated dose has not been established or where the saturation of PK/PD parameters has been observed, favoring PK/PD-guided over fixed-dose recommendations.

## 6. Clinical Implications and Future Directions

The PK variability observed with anti-PD-1 mAbs such as nivolumab, pembrolizumab, and cemiplimab has significant clinical implications. Despite the current regulatory approval of fixed-dose regimens by the FDA and EMA, these strategies may not account for interindividual differences in drug CL and exposure, particularly in long-term responders (patients with ≥6 months of progression-free survival) or patients with altered physiology. As a result, personalized dosing remains underutilized in routine oncology practice.

Emerging PK models, including physiologically based pharmacokinetic (PBPK) simulations and artificial intelligence-driven algorithms, offer promising avenues for individualized treatment. PBPK models can aid in determining CL_0_ and/or ΔCL, complementing Bayesian estimation of PK parameters. Concurrently, machine learning approaches can integrate real-world patient data to refine dose predictions and improve therapeutic outcomes. However, to date, we have not identified any studies applying these technologies in clinical settings.

**Figure 3:**
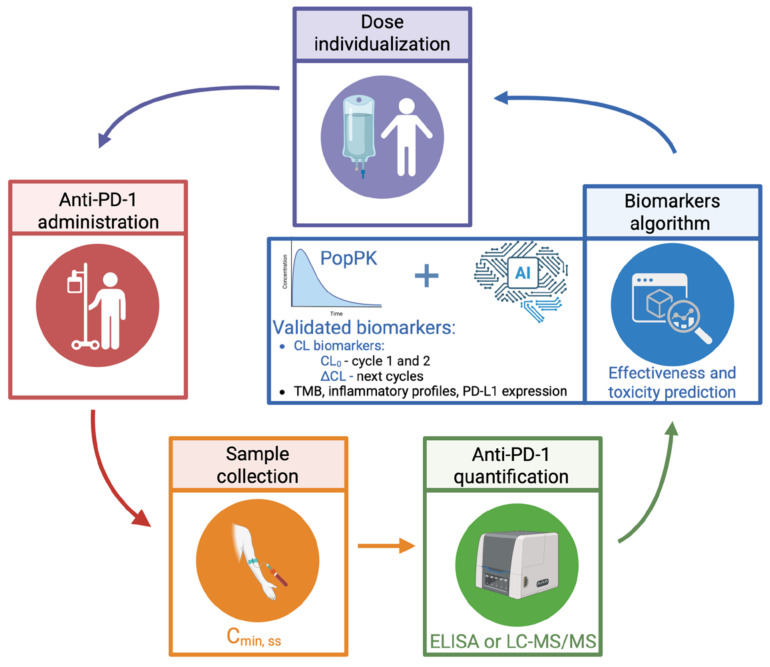
CL-guided dose optimization workflow. Legends: AI—artificial intelligence; CL_0_—basal clearance; ΔCL—change in CL over time; Cmín, ss—steady-state trough concentrations; ELISA—enzyme-linked immunosorbent assay; LC-MS/MS—liquid chromatography tandem mass spectrometry; PopPK—population pharmacokinetic; figure elaborated using BioRender.com.

To translate these innovations into clinical benefit, future research must prioritize the development of real-time PK monitoring tools, as well as large-scale, prospective trials evaluating the safety and efficacy of CL-guided dosing adjustments. Moreover, the integration of PK insights with TDM and validated biomarkers could enhance the precision of immunotherapy and minimize toxicity, while sustaining efficacy. Integrating advanced PK modeling into clinical workflows will be pivotal for the evolution of precision immuno-oncology—Figure 3. However, these strategies face important limitations, including the need for specialized human resources, adequate computational and laboratory equipment, access to drug monitoring data, and validated algorithms, which are not yet available. These efforts, once addressed, promise to personalize treatment strategies, reduce overtreatment, and improve quality of care in patients receiving anti-PD-1 therapies.

Ultimately, an alternative to overcome T cell exhaustion is the combination of anti–PD-1 agents with novel targets under investigation (e.g., anti-TIM-3, anti-TIGIT) [173,174], or strategies aimed at enhancing anti–PD-1 responses, such as co-stimulatory agonists [175], CTLA-4 inhibitors [176,177], or modulation of the tumor microenvironment with repurposed drugs.

## 7. Conclusions

Time-dependent reductions in CL of anti-PD-1 antibodies are consistently associated with improved clinical outcomes but also lead to increased drug exposure, potentially increasing the risk of immune-related toxicities. While standard dosing appears safe across broad populations, individual variability, especially in responders, highlights the need for PK-guided dose optimization. Integrating PK monitoring into clinical practice may enhance efficacy while minimizing toxicity, supporting a more personalized and safer use of anti-PD-1 therapies.

## Figures and Tables

**Table 1:** Anti-PD-1 linear and non-linear pharmacokinetic parameters and models.

Pharmacokinetic	Pembrolizumab [21,23]	Nivolumab [21,24]	Cemiplimab [25]
Model	Two-compartment model with first-order elimination, zero-order iv infusion rate, and time-varying change in CL		
Linear	Doses range: 2–10 mg/kg t_1/2β_ = 22–27.3 days CL = 0.2–0.22 L/day V_dss_ = 6 L	Dose range: 0.1 to 20 mg/kg t_1/2α_ = 32.5 h, t_1/2β_ = 25 days CL = 0.226 L/day V_c_ = 3.63 L; V_p_ = 2.78 L T_máx_ = 1–4 h (Normalized to BW of 80 kg)	Dose range: 1 to 10 mg/kg CL = 0.246 L/day t_1/2β_ = 19.7 days V_dss_ = 5.89 L
Non-linear	Time-varying CL = 16–19 weeks	Time-varying CL = 12–16 weeks	

iv—intravenous; CL—clearance; V_dss_—steady-state volume of distribution; BW—body weight; T_máx_—maximum concentration time; t_1/2α,β_—alfa and beta half-time, respectively.

**Table 2:** Biomarkers of effectiveness and safety.

Biomarker	Category	Status	References
**Effectiveness**			
PD-L1 expression	Protein (tumor/immune)	Approved	[142,143,144]
TMB	Genomic	Approved (TMB ≥10 mut/1 Mb)	[144,145]
dMMR/MSI-H	Genomic	Approved	[146,147,148]
Tumor-infiltrating lymphocytes	Histological	Investigational	[149]
Gene expression signatures	Transcriptomic	Investigational	[135]
Antibiotic use	Clinical factors	Observational	[150]
miRNA signatures (germline)	Epigenetic	Exploratory	[151]
**Effectiveness and/or Safety**			
Lymphocyte counts/NLR	Hematologic	Investigational	[152,153,154]
Circulating cytokines and other inflammatory markers	Soluble proteins	Investigational	[155,156,157]
Clearance monitoring (CL_0_ and ΔCL)	Pharmacokinetics	Investigational	This work * [123]

MSI-H: microsatellite instability; TMB: tumor mutational burden; PD-L1: programmed death-1 ligand; dMMR: DNA mismatch repair; NLR: neutrophil-to-limphocyte ratio; CL_0_—basal clearance; ΔCL—clearance variation by time; * biomarker proposed in the present review; further validation required.

## Abbreviations

The following abbreviations are used in this manuscript:

ADAs	Anti-drug antibodies
ADCC	Antibody-dependent cellular citotoxicity
ALB	Albumin
Anti-TIM3	Anti-T cell mucin domain 3
Anti-TIGIT	Anti-T cell immunoreceptor with immunoglobulin and immunoreceptor tyrosine-based inhibitory motif domains
AUC	Area under plasmatic concentration *versus* time curve
BW	Body weight
CL	Clearance
CL_0_	Baseline clearance
CL–R	Clearance–response
Cmin, ss	Steady-state trough concentrations
CTLA4	Cytotoxic T- lymphocyte-associated antigen 4
DCs	Dendritic cells
ELISA	Enzyme-linked immunosorbent assay
ECM	Extracellular matrix
E–R	Exposure–Response
FcRn	Neonatal Fc receptor
FcRs	Fc receptors
FcγR	Gamma Fc receptor
ICIs	Immune checkpoint inhibitors
IFN-γ	Gamma-interferon
Ig	Immunoglobulin
IL-1	Interleukin 1
irAEs	Immune-related adverse events
LC-MS/MS	Liquid chromatography tandem mass spectrometry
mAbs	Monoclonal antibodies
MMP	Matrix metalloproteinases
NK	Natural killer
NSCLC	Non-small cell lung cancer
OS	Overall survival
PB	Peripheral blood
PBPK	Physiologically based pharmacokinetic
PD	Pharmacodynamics
PD-1	Programmed cell death protein 1
PD-L1	Programmed death-ligand 1
PK	Pharmacokinetics
PK–PD	Pharmacokinetic–Pharmacodynamic
PopPK	Population pharmacokinetics
PS	Performance status
TDM	Therapeutic drug monitoring
TMB	Tumor mutational burden
TMDD	Target-mediated drug disposition
TNF-α	Tumor necrosis factor-alpha
Vd	Volume of distribution
ΔCL	Change in clearance over time
